# Detection of Duffy blood group genotypes and submicroscopic *Plasmodium* infections using molecular diagnostic assays in febrile malaria patients

**DOI:** 10.1186/s12936-024-04875-5

**Published:** 2024-06-20

**Authors:** Beka R. Abagero, Rei Rama, Abdulghani Obeid, Tirusew Tolosa, Biniyam Lukas, Taye Teka, Daniel Tesfaye, Eugenia Lo, Delenasaw Yewhalaw

**Affiliations:** 1 Department of Microbiology and Immunology, Drexel University College of Medicine, Philadelphia, PA USA; 2 Department of Biological Sciences, University of North Carolina at Charlotte, Charlotte, NC USA; 3 School of Medical Laboratory Sciences, Faculty of Health Sciences, Institute of Health, Jimma University, Jimma, Ethiopia; 4 Tropical and Infectious Diseases Research Centre, Jimma University, Jimma, Ethiopia

**Keywords:** Malaria, Submicroscopic *Plasmodium* infection, Microscopy, Quantitative PCR, Duffy genotype, *Plasmodium vivax*, *Plasmodium falciparum*, Ethiopia

## Abstract

**Background:**

Malaria remains a severe parasitic disease, posing a significant threat to public health and hindering economic development in sub-Saharan Africa. Ethiopia, a malaria endemic country, is facing a resurgence of the disease with a steadily rising incidence. Conventional diagnostic methods, such as microscopy, have become less effective due to low parasite density, particularly among Duffy-negative human populations in Africa. To develop comprehensive control strategies, it is crucial to generate data on the distribution and clinical occurrence of *Plasmodium vivax* and *Plasmodium falciparum* infections in regions where the disease is prevalent. This study assessed *Plasmodium* infections and Duffy antigen genotypes in febrile patients in Ethiopia.

**Methods:**

Three hundred febrile patients visiting four health facilities in Jimma town of southwestern Ethiopia were randomly selected during the malaria transmission season (Apr–Oct). Sociodemographic information was collected, and microscopic examination was performed for all study participants. *Plasmodium* species and parasitaemia as well as the Duffy genotype were assessed by quantitative polymerase chain reaction (qPCR) for all samples. Data were analysed using Fisher’s exact test and kappa statistics.

**Results:**

The *Plasmodium* infection rate by qPCR was 16% (48/300) among febrile patients, of which 19 (39.6%) were *P. vivax*, 25 (52.1%) were *P. falciparum*, and 4 (8.3%) were mixed (*P. vivax* and *P. falciparum)* infections. Among the 48 qPCR-positive samples, 39 (13%) were negative by microscopy. The results of bivariate logistic regression analysis showed that agriculture-related occupation, relapse and recurrence were significantly associated with *Plasmodium* infection (*P* < 0.001). Of the 300 febrile patients, 85 (28.3%) were Duffy negative, of whom two had *P. vivax*, six had *P. falciparum*, and one had mixed infections*.* Except for one patient with *P. falciparum* infection, *Plasmodium* infections in Duffy-negative individuals were all submicroscopic with low parasitaemia.

**Conclusions:**

The present study revealed a high prevalence of submicroscopic malaria infections. *Plasmodium* vivax infections in Duffy-negative individuals were not detected due to low parasitaemia. In this study, an improved molecular diagnostic tool was used to detect and characterize *Plasmodium* infections, with the goal of quantifying *P. vivax* infection in Duffy-negative individuals. Advanced molecular diagnostic techniques, such as multiplex real-time PCR, loop-mediated isothermal amplification (LAMP), and CRISPR-based diagnostic methods. These techniques offer increased sensitivity, specificity, and the ability to detect low-parasite-density infections compared to the employed methodologies.

## Background

In malaria-endemic regions where *Plasmodium vivax* and *Plasmodium falciparum* coexist, *P. vivax* continues to be the main cause of malaria because existing interventions are primarily focused on *P. falciparum*. *Plasmodium vivax* causes severe and fatal outcomes that have reversed the historical notion of benign *P. vivax* infections[1]. However, despite this burden, *P*. *vivax* does not draw as much attention as *P. falciparum,* particularly in sub-Saharan Africa [2].

Malaria is a major public health problem in Ethiopia, of which 75% of the landmass of the country is endemic to malaria. The transmission of malaria occurs throughout the year, but it varies based on altitude and seasonality. In Ethiopia, malaria accounts for approximately 60% of all hospital admissions. Many malaria-related illnesses and deaths occur in the most remote, rural areas of the country where there is insufficient health care coverage, poverty, and other socioeconomic issues [3–5]. Ethiopia is one of the few African countries where *P. falciparum* and *P. vivax* coexist [6, 7]. *Plasmodium falciparum* accounted for 70% of all malaria cases, and the remaining cases were attributed to *P. vivax* [8, 9]. Efforts to control malaria in Ethiopia have made significant progress in recent years. Between 2015 and 2020, malaria cases decreased by approximately 39%, and malaria-related deaths decreased by approximately 34%. While malaria prevalence has shown a slight decline in the past few years, transmission appears to be heterogeneous across the country, with some areas still facing a high malaria burden [10].

The malaria situation has changed since the COVID-19 pandemic, and such impact varies among nations due to the country's unique epidemiological context, size, and intervention coverage. Based on recent geospatial estimates, the disruption caused by COVID-19 to malaria control in Africa resulted in almost a doubling of malaria mortality in 2020 compared to previous years. Furthermore, this disruption might lead to even more significant increases in subsequent years if not properly addressed [11, 12].

Malaria control and elimination strategies heavily depend on timely, accurate diagnosis and effective treatment [13, 14]. Microscopy is a gold standard and common malaria diagnostic test in several African countries because of its high specificity, convenience with a rapid turnaround time, and low cost. However, it has limited sensitivity, especially for low parasitaemia infections, which require highly skilled microscopists complemented by molecular assays. An improved point-of-care detection method is critical because transmission caused by low parasitaemia infections hinders the progress and goal of malaria elimination [13]. Systematic diagnosis and treatment of individuals with submicroscopic *Plasmodium* infections as part of the surveillance and intervention strategy would reduce and eliminate the parasite reservoirs that sustain transmission [14, 15].

Another factor that impacts malaria infection is Duffy blood antigens. The Duffy antigen receptor for chemokines (DARC) is encoded by the *DARC* gene expressed on the surface of red blood cells (RBCs) and plays a role in RBC invasion by the *P. vivax* parasite [16, 17]. Individuals who lack Duffy antigen expression (Duffy-negative individuals) are known to be resistant to *P. vivax* [18, 19] and have a reduced risk of *P. vivax* infection [20, 21]. However, Duffy negativity does not provide complete protection against vivax malaria [22]. Other *Plasmodium* species, such as *P. falciparum,* can also infect and cause malaria in Duffy-negative individuals. Additionally, Duffy-negative individuals can be low-parasitaemia carriers [23, 24]. The parasites remain dormant in the liver and cause relapses later, which can sustain transmission and hinder complete elimination of the disease [25–27].

With the goal of eliminating malaria in Ethiopia and other parts of SSA, there is a pressing need for accurate diagnosis and effective treatment of low density parasitaemia *Plasmodium* infection in Duffy-negative Individuals. Understanding the distribution and prevalence of *Plasmodium* infections can contribute to better implementation of control strategies. This study assessed the performance of molecular assays to detect *Plasmodium* infection, especially to detect infections with low parasitaemia in Duffy-negative individuals, which can hinder progress toward malaria elimination. The findings can aid policymakers and programmes in designing more effective and targeted interventions to combat malaria and work toward its ultimate elimination in the region.

## Methods

### Study site and sample collection

**Fig. 1:**
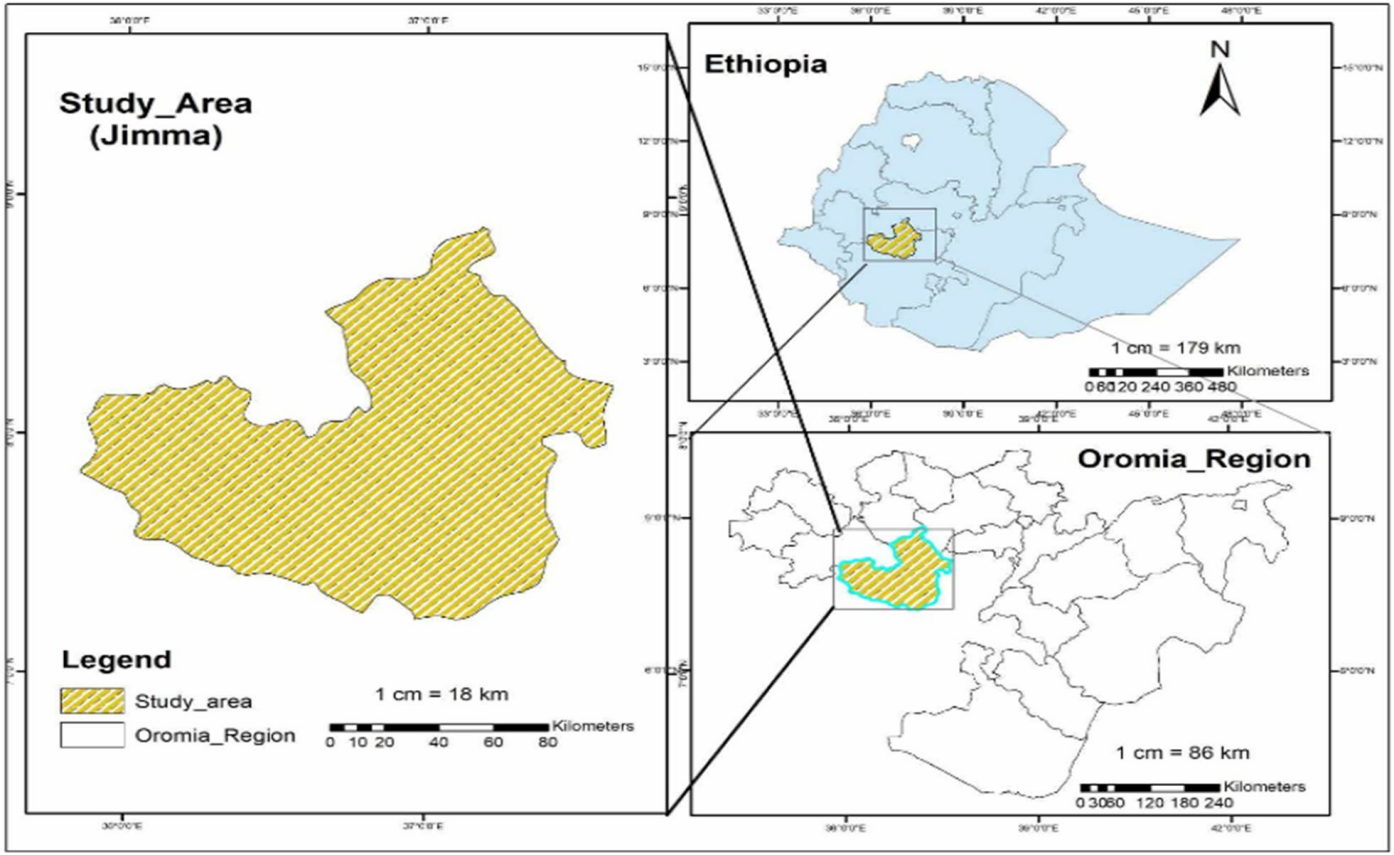
Map of the study area

A total of 300 samples were collected from febrile patients from three health facilities (Jimma Shene Gibe General Hospital, Jimma Higher One Health Centre, and Jimma Higher Two Health Centre of Jimma town), southwestern Ethiopia. The area is located at Latitude: 7°40'N and Longitude: 36°50′ E with an altitude of 1780 m; (Fig. 1). The study was conducted from May to October 2022, and the study design was a cross-sectional study design. Sociodemographic data, including gender, age, occupation, education, and ethnicity, were collected from each study participant. Previous malaria history, anti-malarial drug treatment, medical history, and Duffy status were collected for malaria infection risk analysis. For participants aged below 18 years old, the questionnaire was completed by their guardians or parents. A finger-pricked blood sample of approximately 200 µl was collected and preserved on Whatman filter paper [28]. Thin and thick blood smears were prepared for microscopic examination of malaria parasites [29].

### Blood film preparation and slides examination

Thin and thick blood films were prepared on slides for each study subject, following a standard protocol [30]. The blood smears were air-dried, fixed with absolute methanol, and stained with 10% Giemsa at the hospital for malaria parasite detection and quantification. The stained slides were examined under a microscope using 100 × oil immersion. A blood smear slide was labelled "positive" for malaria if parasites were detected, and "negative" if no parasites were observed after examining 200 oil immersion fields of the thick smear by following the standard World Health Organization (WHO) protocol [31, 32]. When parasites were found, their density was determined by counting the number of parasites against 200 white blood cells in the thick smear, assuming an average WBC count of 8000/µl of blood [33]. The thin smears were used to identify the species of *Plasmodium*. Both parasite species and density were recorded. All blood films were first read on site or at hospital laboratories by trained lab technologists. A subset, including films positive for parasites and 10% of negative slides, were re-examined by blinded laboratory technologists at the JU-TIDRC research laboratory for validation.

### Parasite DNA extraction and molecular screening

For each sample, parasite DNA was extracted from a dried blood spot (~ 50 µl) using the Saponin/Chelex method [34]. *Plasmodium* species were examined by quantitative PCR of the 18S rDNA gene using species-specific primers for *P. falciparum* and *P. vivax* [35, 36]. Amplification was performed in a 20 μl reaction mixture containing 2 μl of genomic DNA, 10 μl 2 × SYBR Green qPCR Master Mix (Thermo Scientific), and 0.5 µM primer with an initial denaturation at 95 °C for 3 min, followed by 45 cycles at 94 °C for 30 s, 55 °C for 30 s, and 68 °C for 1 min with a final 95 °C for 10 s. This was followed by a melting curve step of temperature ranging from 65 °C to 95 °C with 0.5 °C increments to determine the melting temperature of each amplified product. Each assay included positive controls of *P. falciparum* (MRA-667) and *P. vivax* (MRA-178) strains, in addition to negative controls including uninfected samples and nuclease-free water. A standard curve was generated from a tenfold serial dilution of the control plasmids to determine the efficiency of qPCR. Melting curve analyses were performed for each amplified sample to confirm specific amplifications of the target sequence. Samples yielding a threshold cycle (Ct value) higher than 40 (as indicated in the negative controls) were considered negative for *Plasmodium* species. Parasite density in a sample was quantified with the following equation: GCN sample = 2^E×(40−Ct sample)^, where GCN stands for gene copy number, Ct for the threshold cycle of the sample, and E for amplification efficiency [37]. The differences in the log-transformed parasite GCN between the microscopic-positive and microscopic-negative samples were assessed for level of significance at *p* < 0.05 by one-tailed t test.

### DARC genotyping

**Fig. 2:**
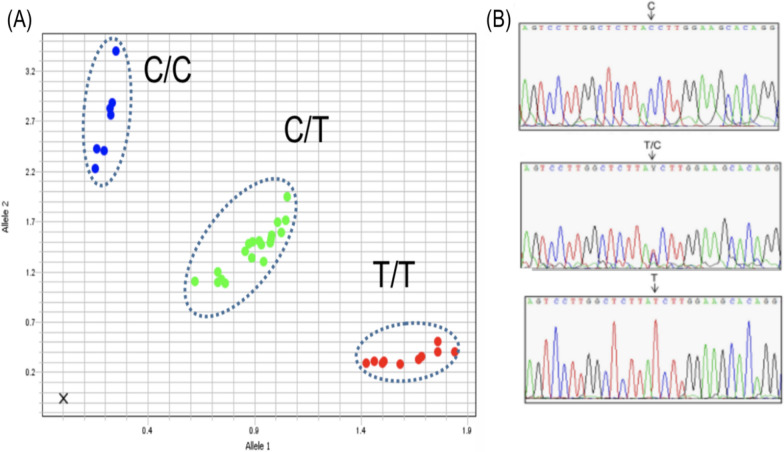
Allelic discrimination plot to distinguish Duffy genotypes. **A** TaqMan-based allelic discrimination plot showing C/C (Duffy-negative), C/T and T/T genotypes. **B** *DARC* sequence by Sanger confirming the TaqMan genotype

An approximately 500-bp fragment of the human DARC gene that encompasses the SNP position rs2814778 (− 67T > C) located in the promoter region was amplified following established protocols[38]. Amplifications were conducted in a mixture containing 2 μl of genomic DNA, 7 μl of Taqman Fast Advanced Master Mix (Thermo Fisher), 0.54 µM of each forward and reverse primer and 0.54 µM of each Probe C-FAM and T-HEX. The reactions were performed with an initial denaturation at 94 °C for 2 min, followed by 35 cycles at 94 °C for 30 s, 58 °C for 30 s, and 65 °C for 40 s, with a final 2-min extension at 65 °C. An allelic discrimination plot was used to distinguish Duffy genotypes. DARC genotypes were confirmed by PCR and Sanger sequencing for all Duffy-negative and a subset of Duffy-positive samples (Fig. 2).

### Data analyses

All data were analysed using SPSS software package version 21.0. Descriptive statistics were used to summarize the sociodemographic characteristics of the study participants. Bivariate logistic regression was used to determine the association of malaria infection with independent variables. The odds ratio and the corresponding 95% CI were calculated to determine the strength of the association. A *p* value < 0.05 was considered statistically significant during the analysis.

## Results

### Demographic and socioeconomic factors

Of the 300 study participants, 126 (42.0%) were males and 174 (58.0%) were females (Table 1). The median age of the participants in the study was 24 years (range = 1–85 years). Approximately 29% (*n* = 86) of the participants were engaged in agriculture and open field work (farmers, construction workers, guardsmen, soldiers, and gardeners), and the remaining (*n* = 214, 71.3%) were office clerks, teachers, shopkeepers, full-time students, preschool children, and housekeepers (Table 1). The results of bivariate logistic regression analysis showed that agriculture-related occupation, relapse and recurrence were significantly associated with *Plasmodium* infection (*P* < 0.001). Individuals with a history of malaria infection who failed to comply with the complete anti-malarial drug prescription protocol were found to be at a higher risk of *Plasmodium* infections. Conversely, those who diligently adhered to the complete course of anti-malarial drugs prescribed were found to be at a lower risk of *Plasmodium* infection (OR: 0.3; CI: 0.1–0.72; *P* = 0.009; Table 1). Individuals who had agriculture-related occupations were 27-fold at higher risk of malaria infection than individuals who were engaged in other occupations (OR: 27.36; CI: 9.3–80.8; *P* < 0.001; Table 1). The differentiation was based on patient self-reporting during the questionnaire, with subsequent confirmation via medical records where available. Relapse was defined as a new episode of malaria after treatment without re-exposure, while recurrence was defined as a new episode following re-exposure. The limitations of the self-reported data are duly acknowledged in this study.

**Table 1:** Demographic characteristics and Risk factors associated with clinical malaria among malaria suspected individuals in Ethiopia

Parameters	Number of samples	Total infection	Odds ratio (95% CI)	*P. vivax*	Odds ratio (95% CI)	*P. falciparum*	Odds ratio (95% CI)	Mixed infection	Odds ratio (95% CI)
Overall									
	300	48 (16%)		19 (40%)		25 (52.1%)		4 (8.3%)	
Gender									
Female	174 (58%)	23	1	9	1	12	1	4	1
Male	126 (42%)	25	1.62 [0.9–3.] P = 0.124	10	1 [0.32–3.3] P = 0.950	13	0.7 [0.2–2.34] P = 0.552	2	0.7 [0.1–3.8] P = 0.671
Age									
≥ 4 years	31 (10.3%)	7	1	1	1	6	1	0	1
≤ 5 to ≥ 15	77 (25.6%)	11	1.8 [0.61–5] P = 0.299	5	2.1 [0.23–18.6] P = 0.511	6	0.8 [0.1–9.2] P = 0.861	0	
≥ 16 to ≥ 64	182 (61%)	29	1.53 [0.6–3.9] P = 0.364	13	2.3 [0.3–18.3] P = 0.428	12	1.5 [0.2–12.5] P = 0.690	6	0.9 [0.04–18.4] P = 0.924
≥ 65	10 (3.3%)	1	2.62 [0.3–24.43] P = 0.396	0	3.3 [0.2–58.8] P = 0.411	1	1.0 [0.03–26.5] P = 1.00	0	
Educational status									
Educated^a^	249 (83%)	29	1	14	1	19	1	5	1
Non-Educated^b^	51 (17%)	7	0.82 [0.34–2] P = 0.677	5	1.82 [0.6–5.3] P = 0.270	6	0.44 [0.1–3.5] P = 0.442	1	1 [0.04–20.5] P = 0.983
Primary occupation									
Agricultures related	85 (28.3%)	37	27.4 [9.3–80.8] P = 0.0001	17	18 [4.0–80.1] P = 0.0001	13	0.6 [0.14–2.4] P = 0.446	7	1.4 [0.1–23.6] P = 0.817
In door	146 (49%)	4	1	2	1	1	0.3 [0.02–2.6] P = 0.245	3	3.6 [0.2–66.2] P = 0.389
Outdoor	67 (22.3%)	4	2.3 [0.54–9.3] P = 0.261	2	2 [0.3–16.1] P = 0.431	2	1	0	1
Have you been diagnosed for malaria in the past (if yes)									
No	168 (56%)	22	1	14	1	7	1	1	1
0–3 months ago	17 (6%)	2	0.9 [0.2–4.13] P = 0.876	1	0.6 [0.1–4.5] P = 0.580	0	0.6 [0.03–11.7] P = 0.764	1	10 [0.6–165.2] P = 0.110
4–6 months ago	67 (22.3%)	8	0.9 [0.4–2.13] P = 0.810	5	0.9 [0.3–2.6] P = 0.825	3	1.1 [0.3–4.3] P = 0.915	0	3.2 [0.1–81.8] P = 0.480
7–11 months ago	20 (6.6%)	2	0.73 [0.2–3.4] P = 0.696	1	0.7 [0.1–5.7] P = 0.327	1	1.4 [0.2–12.2] P = 0.753	0	3.2 [0.1–81.8] P = 0.480
≥ 1 years ago,	28 (9.2%)	2	0.51 [0.11–2.3] P = 0.381	1	0.7 [0.1–5.7] P = 0.327	1	1.4 [0.2–12.2] P = 0.753	0	3.2 [0.1–81.8] P = 0.480
Did you take all anti-malaria drugs in the previous prescriptions									
Yes	25 (8.3%)	9	1	3	1	6	1		
No	100 (33.3%)	13	0.3 [0.1–0.72] P = 0.009	5	0.4 [0.1–1.73] P = 0.214	8	1 [0.1–9.34] P = 1.00		
Previous malaria infection									
No	168 (56%)	22	1	11	1.4 [0.6–3.4] P = 0.487	11	1.1 [0.34–3.54] P = 0.873		
Yes	132 (44%)	22	1.32 [0.7–2.51] P = 0.386	8	1	14	1		
Duffy status									
CT	215 (72%)	38	1.81 [0.8–3.9] P = 0.132	17	3.6 [0.8–15.8] P = 0.094	19	1.3 [0.5–3.31] P = 0.61	4	0.9 [0.23–3.6] P = 0.908
TT									
CC	85 (28.3%)	9	1	2	1	6	1	1	1
Body temperature									
Normal (≤ 37.5)	263 (88%)	37	1	19	1	20	1	6	
Febrile (> 37.5)	37(12.3%)	4	0.74 [0.24–2.21] P = 0.590	0	0.83 [0.2–3.8] P = 0.815	5	0.54 [0.1–4.3] P = 0.566	0	3.6 [0.2–66.2] P = 0.389

### *Plasmodium* detection in Duffy-negative patients by microscopy and qPCR

The frequencies of Duffy-negative and Duffy-positive genotypes among all participants were 85 (28.3%) and 215 (71.6%), respectively. These findings underscore the importance of the Duffy blood group in regard to malaria susceptibility and other health outcomes. Specifically, 41 males and 44 females were found to be Duffy negative, while 85 males and 130 females were Duffy positive, indicating a higher proportion of females in this group. These results suggest that further research on the relationship between the Duffy blood group and health outcomes is needed to better understand the implications of these findings. Both *P. vivax* and *P. falciparum* infections were observed in Duffy-negative patients (Table 2). This study revealed that Duffy-positive individuals were at higher risk of having *P. vivax* than Duffy-negative individuals (OD: 7.65 [1.37–42.7] *P* = 0.020 and OD: 6.18 [1.1–34.7] *P* = 0.038, respectively) (Table 2). However, no such association was found for *P. falciparum* or mixed (*P. vivax* and *P. falciparum*) infections among the different Duffy genotypes.

**Table 2:** The distributions of malaria cases by Duffy genotype using qPCR among malaria-suspected individuals in Ethiopia

Malaria species	Total number of infections	Duffy genotype				
		Hemizygous infected (CT)	Odd ratio (95% CI) (CC vs. CT)	Homozygous infected (TT)	Odd ratio (95% CI) (CC vs. TT)	Homozygous infected (C/C)
Pv	19	9	7.65 [1.37–42.7] P = 0.020	8	6.18 [1.10–34.70] P = 0.038	2
Pf	25	10	2.11 [0.62–7.13] P = 0.229	9	1.78 [0.52–6.08] P = 0.357	6
Mixed	4	2	3.00 [0.15–59.89] P = 0.472	1	1.00 [0.04–24.54] P = 1.000	1
Total	48	21		18		9

### Prevalence of submicroscopic infections

A higher number of malaria-positive samples were detected by qPCR (*n* = 48; 16.0%) than by microscopy (*n* = 29; 9.6%; Table 3). Among qPCR-positive samples, 19 (39.58%) had *P. vivax*, 25 (52.08%) had *P. falciparum*, and 4 (8.33%) had mixed infections (*P. falciparum* and *P. vivax)*. Interestingly, only 4 out of 19 *P. vivax* samples and 3 out of 25 *P. falciparum* samples were identified by microscopy from qPCR-positive samples. No mixed infections were identified (Table 3). Moreover, *P. falciparum* and *P. vivax* infections were confirmed by qPCR (n = 39, 13%) to be negative by microscopy and were submicroscopic *Plasmodium* infections. The sensitivity of microscopy can vary based on the skill of the technician and the quality of the microscope and slides. It is generally less sensitive in detecting low levels of parasitaemia because the parasites might be too few to be seen within the examined fields of the blood smear.

**Table 3:** Prevalence of submicroscopic and missed infections among malaria-suspected individuals in Ethiopia

qPCR		Microscopy			
		Pv	Pf	Mixed	False negative
		15	14	0	
Pv	19	4	0	0	15
Pf	25	1	3	0	21
Mixed	4	1	0	0	3
Sub-microscopy and miss diagnosed					39

### Duffy genotype and *Plasmodium* parasitaemia density

**Fig. 3:**
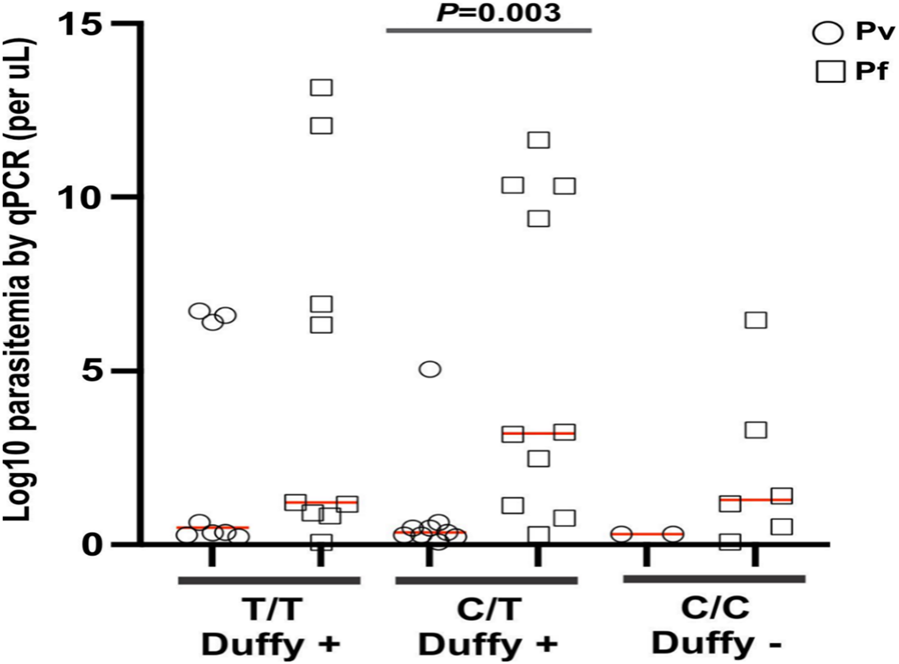
Dot plots of the Duffy genotype by log-transformed parasite gene copy number of Plasmodium-infected samples

Figure 3 presents the distribution of the Duffy genotype in relation to parasite density. Overall, a significant difference was observed in the parasite density among the Duffy phenotypes (p < 0.05). Notably, individuals with the Duffy-positive phenotype (C/T) exhibited significantly higher *P. falciparum* parasite density than individuals with Duffy-negative phenotypes (CC, T/T) (p < 0.003). The study finds no statistically significant difference in *P. vivax* densities among different Duffy phenotypes.

## Discussion

The Ethiopian government aims to eliminate malaria by 2030. However, submicroscopic infections, relapses of *P. vivax*, and residual transmission present tremendous challenges to this goal. Submicroscopic infections contribute to malaria transmission. The prevalence of submicroscopic malaria infections detected in this study was higher than that previously reported in low-endemic areas such as Malo (9.7%) and Bonga (10%) of southwest Ethiopia [39] but lower compared to regions in southwestern Saudi Arabia, Senegal, and Uganda, where submicroscopic malaria infections were more than 23% [40–42]. Infected individuals with submicroscopic parasitaemia have the potential to serve as infection reservoirs in the community and other malaria-free areas as these infections are not detected and treated with anti-malarial drugs [39].

Failure to detect submicroscopic infections, especially in the case of *P. vivax* in Duffy-negative individuals, poses a significant challenge to malaria control and elimination efforts. These infections can remain unnoticed and untreated, leading to persistent and ongoing transmission in the affected communities. Duffy-negative individuals can serve as parasite reservoirs for new infections to emerge and circulate in Duffy-positive individuals [43]. This study indicated that 28% of the febrile patients were Duffy negative. This proportion is slightly higher than an earlier report of ~ 20% homozygous Duffy-negative patients in Harar and Jimma [44], but was much lower than the proportion of Duffy-negative patients in West and Central Africa (> 97%) [45, 46]. Surveillance that relies on detecting symptomatic cases and carrying out routine blood smears or RDTs might underestimate the true prevalence of *P. vivax* in Duffy-negative individuals. Duffy-negative individuals can be infected with *P. vivax*, as well as other malaria parasite species such as *P. falciparum* as mono- or mixed-infections [47]. Outside Africa, in the Brazilian Amazon, a *P. vivax* endemic area, a small percentage of Duffy-negative individuals have been infected with *P. vivax* [48], suggesting that alternative invasion pathways may exist for this parasite. Mixed infections involving *P. vivax* and *P. falciparum*, as well as other mixed infections of malaria parasite species, were detected in both Duffy-positive and Duffy-negative individuals [49].

Figure 3 shows that the Duffy genotype is significantly associated with the density of *P. falciparum* parasites in individuals, with Duffy-positive individuals having higher densities. However, this association is not observed with *P. vivax*. This could have important implications for understanding malaria infection dynamics and potential treatments or interventions. Given that *P. vivax* infections in Duffy-negative individuals have low parasite densities, a sensitive and molecular diagnostic assay such as qPCR is needed to accurately detect these infections and compare *P. vivax* epidemiology in malaria endemic countries. In the case of *P. falciparum*, there is evidence to suggest that individuals with the Duffy-negative genotype may have some level of protection against this parasite [50]. *Plasmodium falciparum* is known to use different receptors for invasion, and it is less dependent on the Duffy antigen compared to *P. vivax.* Therefore, individuals with the Duffy-negative genotype may be less susceptible to *P. falciparum* infection. It is important to note that the relationship between Duffy genotypes and malaria susceptibility can vary among different populations, and other genetic and environmental factors also play a role in determining susceptibility to malaria. Additionally, research in this field is ongoing, and new findings may contribute to a deeper understanding of the complex interactions between host genetics and malaria infection.

It is not surprising that individuals who had a previous history of malaria infection with not taking all anti-malarial drugs in the previous prescriptions had a significantly higher risk for malaria infection than those who took all anti-malarial drugs in the previous prescriptions. This study provides compelling evidence that the complete course of anti-malaria drugs prescribed should be diligently followed to reduce the risk of malaria infection. The risk of relapse or reinfection could be affected by the complex immune responses developed after an initial infection. When a person is infected with malaria, the immune system produces specific antibodies and immune cells that target the parasites. While such immune responses recognize and eliminate parasite strains that cause the initial infection [51], some strains may evade the immune system and remain dormant in the liver [52]. These parasites can persist in the body at low levels, leading to a chronic low-grade infection. In individuals who have had a previous malaria infection, the presence of residual parasites can lead to a faster and more robust immune response upon reinfection. This phenomenon is known as a "premunition" response [53]. The high levels of antibodies and immune cells generated in response to the first infection can sometimes contribute to an exaggerated immune response, leading to increased inflammation and tissue damage in subsequent infection. The duration of dormancy varies depending on the parasite strain, but it is generally thought to be several months [54]. Therefore, individuals who have experienced a recent malaria infection, particularly within the previous 4–6 months, are more likely due to dormant parasites in the liver. This increases their susceptibility to reinfection compared to individuals who have never been infected or have a more distant history of infection. It is important to note that the risk of malaria infection can also depend on the complex interactions between the immune system and the characteristics of malaria parasites, including its ability to persist at low levels, evade immune detection, specific strain of the parasites, the intensity of malaria transmission in a given area, the use of preventive measures such as bed nets or insecticide spraying, and the individual's overall health and immune status. These factors can further influence the risk of malaria reinfection in individuals with a previous infection history.

## Conclusion

Submicroscopic malaria infections contribute to persistent transmission of the disease. The present study revealed that submicroscopic infection in Duffy-negative individuals was high. Microscopy failed to detect a significant number of *Plasmodium* infections in Duffy-negative individuals. However, molecular diagnostic assays such as qPCR could detect more submicroscopic infections in Duffy-negative individuals than microscopy. This study supports the notion that *P. vivax* can infect Duffy-negative individuals. Therefore, improved diagnostic methods are critical to detect submicroscopic *Plasmodium* infections in Duffy-negative individuals. This could reduce reservoir infections, which enhances malaria elimination efforts.

## Data Availability

The data related to this research can be obtained from the corresponding author upon reasonable request.

## Abbreviations

COR: Crude odds ratio
GCN: Gene copy number
ITN: Insecticide treated net
qPCR: Quantitative polymerase chain reaction
RDT: Rapid diagnostic tests
SPSS: Statistical package for social sciences
SSA: Sub-Saharan Africa
WHO: World Health Organization
